# [Retracted] MicroRNA‑203 inhibits the proliferation and invasion of U251 glioblastoma cells by directly targeting PLD2

**DOI:** 10.3892/mmr.2023.13154

**Published:** 2023-12-27

**Authors:** Zigui Chen, Dazhi Li, Quan Cheng, Zhiming Ma, Bing Jiang, Renjun Peng, Rui Chen, Yiqiang Cao, Xin Wan

Mol Med Rep 9: 503–508, 2014; 10.3892/mmr.2013.1814

Following the publication of this paper, it was drawn to the Editors’ attention by a concerned reader that the PLD2 western blotting data shown in Fig. 3A and the Transwell invasion assay data shown in Fig. 6 were strikingly similar to data appearing in different form in other articles written by different authors at different research institutes that had either already been published elsewhere prior to the submission of this paper to *Molecular Medicine Reports*, or were under consideration for publication at around the same time. In view of the fact that certain of these data had already apparently been published previously, the Editor of *Molecular Medicine Reports* has decided that this paper should be retracted from the Journal. The authors were asked for an explanation to account for these concerns, but the Editorial Office did not receive a reply. The Editor apologizes to the readership for any inconvenience caused.

